# Bridging Rare and Abundant Bacteria with Ecosystem Multifunctionality in Salinized Agricultural Soils: from Community Diversity to Environmental Adaptation

**DOI:** 10.1128/mSystems.01221-20

**Published:** 2021-03-30

**Authors:** Wenjie Wan, Song Liu, Xiang Li, Yonghui Xing, Wenli Chen, Qiaoyun Huang

**Affiliations:** a State Key Laboratory of Agricultural Microbiology, Huazhong Agricultural University, Wuhan, People’s Republic of China; b Key Laboratory of Arable Land Conservation (Middle and Lower Reaches of Yangtze River), Ministry of Agriculture, Huazhong Agricultural University, Wuhan, People’s Republic of China; c Key Laboratory of Aquatic Botany and Watershed Ecology Wuhan Botanical Garden, Chinese Academy of Sciences, Wuhan, People’s Republic of China; Lawrence Berkeley National Laboratory

**Keywords:** bacterial diversity, environmental breadth, phylogenetic signal, phylogenetic clustering, salinity, stochastic versus deterministic

## Abstract

Bacterial diversity and ecosystem multifunctionality (EMF) vary along environmental gradients. However, little is known about interconnections between EMF and taxonomic and phylogenetic diversities of rare and abundant bacteria. Using MiSeq sequencing and multiple statistical analyses, we evaluated the maintenance of taxonomic and phylogenetic diversities of rare and abundant bacteria and their contributions to EMF in salinized agricultural soils (0.09 to 19.91 dS/m). Rare bacteria exhibited closer phylogenetic clustering and broader environmental breadths than abundant ones, while abundant bacteria showed higher functional redundancies and stronger phylogenetic signals of ecological preferences than rare ones. Variable selection (86.7%) dominated rare bacterial community assembly, and dispersal limitation (54.7%) and variable selection (24.5%) determined abundant bacterial community assembly. Salinity played a decisive role in mediating the balance between stochastic and deterministic processes and showed significant effects on functions and diversities of both rare and abundant bacteria. Rare bacterial taxonomic α-diversity and abundant bacterial phylogenetic α-diversity contributed significantly to EMF, while abundant bacterial taxonomic α-diversity and rare bacterial phylogenetic α-diversity did not. Additionally, abundant rather than rare bacterial community function had a significant effect on soil EMF. These findings extend our knowledge of environmental adaptation of rare and abundant bacteria and highlight different contributions of taxonomic and phylogenetic α-diversities of rare and abundant bacteria to soil EMF.

**IMPORTANCE** Soil salinization is a worldwide environmental problem and threatens plant productivity and microbial diversity. Understanding the generation and maintenance of microbial diversity is essential to estimate soil tillage potential via investigating ecosystem multifunctionality. Our sequence-based data showed differences in environmental adaptations of rare and abundant bacteria at taxonomic and phylogenetic levels, which led to different contributions of taxonomic and phylogenetic α-diversities of rare and abundant bacteria to soil EMF. Studying the diversity of rare and abundant bacteria and their contributions to EMF in salinized soils is critical for guiding soil restoration.

## INTRODUCTION

The ultimate purpose of agricultural soil study is to serve human society by cultivating plants to supply people with products (e.g., food, fiber, and rubber) (1). Evaluating the tillage potential of agricultural soils by evaluating productivity is challenging when the plant shows the wrong fitness for the soil type (2). Soil ecosystem multifunctionality (EMF), regarded as an important indicator for estimating plant diversity and nutrient cycling and retention (3), makes it possible to assess soil tillage potential. Soil EMF is reported to be closely positively correlated with biotic, especially microbial, α-diversity (4–6). Microorganisms, particularly bacteria, are responsible for soil key nutrient cycling, including carbon fixation and degradation, nitrogen fixation and nitrification, and phosphorus mineralization and solubilization (7–9). However, microbial diversity is determined by complex environmental conditions (e.g., salinity and pH) (10, 11). Understanding microbial diversity in different habitats is important for predicting soil EMF. However, the relationship between microbial diversity and EMF in salinized agricultural soils is poorly investigated.

Soil salinization, regarded as a “hidden killer of agriculture,” is a globally agricultural problem threatening crop growth and productivity (12). Saline soils, defined as soils with an electrical conductivity (EC) in a saturated soil extract of >4 dS/m, are distributed globally in arid and semiarid regions (11). Traditional approaches, including soil respiration, microbial growth rate, microbial biomass, and enzymatic activity, have revealed that microbes exhibit different levels of salt tolerance in different sites (13–15). Next-generation sequencing technology has clarified that both diversity and community composition of bacteria vary in different salinity gradients in different terrestrial ecosystems (11, 16–18). Divergences in environmental stress caused by different salinity ranges might lead to differences in growth, activity, diversity, and composition of the bacterial community (11, 13). This can result in a skewed distribution of microbial abundance in a local microbial community, with relatively few dominant species and a large number of rare species (alternatively known as a “rare biosphere”) (19). Prior studies have reported that rare and abundant microbial taxa often show distinct distribution patterns and functional traits in agricultural soils (20, 21). However, the effects of salinity on the distribution of rare and abundant microbial species and the functions they provide are rarely reported. Therefore, elucidating the distribution pattern and community assembly mechanisms of rare and abundant microbial taxa is critical for understanding diversity-driven ecosystem processes and functions.

The abundance of a rare or abundant species is the consequence of a balance between its growth and death rates (22). Rare and abundant species exhibit diverse responses to environmental changes (21, 23). For instance, the community composition of abundant bacteria is more affected by environmental factors than is that of rare bacteria in Tibetan Plateau grassland soils (24). Environmental thresholds of bacteria in response to nitrogen addition in a semiarid steppe are assessed by applying the accumulated values of change points of all of the species in the tested bacterial community (25). In addition, the responses of microorganisms to environmental changes show phylogenetic conservatism, and in this case, microorganisms are not distributed randomly across the tree of life (26). For example, the carbon utilization efficiencies of 23 soil bacteria isolates show strong conservatism in response to temperature (27). Environmental filtering of salinity and pH are crucial determinants in shaping microbial diversity and community composition (28, 29); the response traits of salinity and pH preference of microorganisms are reported to be deeply conserved at the phylogenetic level (26). For instance, in *Acidobacteria*, salinity preference appears to be deeply conserved at the phylum level. Therefore, estimating the phylogenetic patterns of microbial response traits provides predictions for microbial distribution pattern and their responses to environmental change. However, the response threshold and phylogenetic pattern of the bacterial community to ongoing environmental change, especially rare and abundant taxa, have rarely been studied in salinized agricultural soils.

Ecological community assembly processes determine microbial community structure and co-occurrence and compulsorily couple the composition of a microbial community with the functions the microbes supply (30–32). Generally, stochastic (e.g., dispersal limitation and homogenizing dispersal) and deterministic (e.g., homogeneous selection and variable selection) processes have been evaluated with respect to their contributions to microbial community assembly (33, 34). Consequently, the rare microbial community is more strongly affected by stochasticity than the abundant one in Tibetan Plateau grassland soils (24), in a salt marsh ecosystem (35), and in rice paddy soils (36). Environmental factors play important roles in balancing stochastic and deterministic processes for microbial community assembly (20, 34, 37). For example, soil organic matter shows a strong effect on bacterial community assembly (31). Yet, it remains unclear whether similar environmental variables mediate the balance between stochastic and deterministic processes in community assembly of rare versus abundant bacteria in salinized soils.

Chinese farmlands, responsible for feeding 1.4 billion people, have to provide agriculture products as much as possible via applying fertilizers. However, the excessive application of inorganic fertilizer leads to soil salinization, and low precipitation and poor agricultural management intensify salinization (13, 17, 18). This situation triggered our thinking and motivated us to investigate the tillage potential of salinized agricultural soils by determining soil EMF. In view of this, we selected a representative area in Yingcheng City (Hubei Province, China) and collected 90 soil samples suffering from different degrees of salinization (0.09 to 19.91 dS/m). In the present study, we aimed to (i) investigate relationships between soil EMF and rare and abundant bacterial diversities in salinized agricultural soils, (ii) evaluate environmental adaptation of rare and abundant bacterial taxa, and (iii) reveal community assembly processes of rare and abundant bacteria. Considering low competition potential and growth rate of rare taxa (38, 39), we hypothesized that rare microbial taxa would exhibit narrower environmental thresholds and weaker phylogenetic signals for traits than abundant microbial taxa. Because soil EMFs are closely correlated with both taxonomic (4, 5) and phylogenetic (6) α-diversities, we also hypothesized that there would be close links between EMF and taxonomic and phylogenetic α-diversities of rare and abundant bacteria. To achieve our goals and validate our hypothesis, we applied Illumina MiSeq sequencing for the bacterial 16S rRNA gene and characterized soil physicochemical properties and enzyme activities. We found distinct differences in distribution patterns, levels of phylogenetic clustering, environmental thresholds, phylogenetic signals, and community assembly processes between rare and abundant bacteria.

## RESULTS

### General distribution characteristics, diversities, and functions of rare and abundant bacteria.

**FIG 1:**
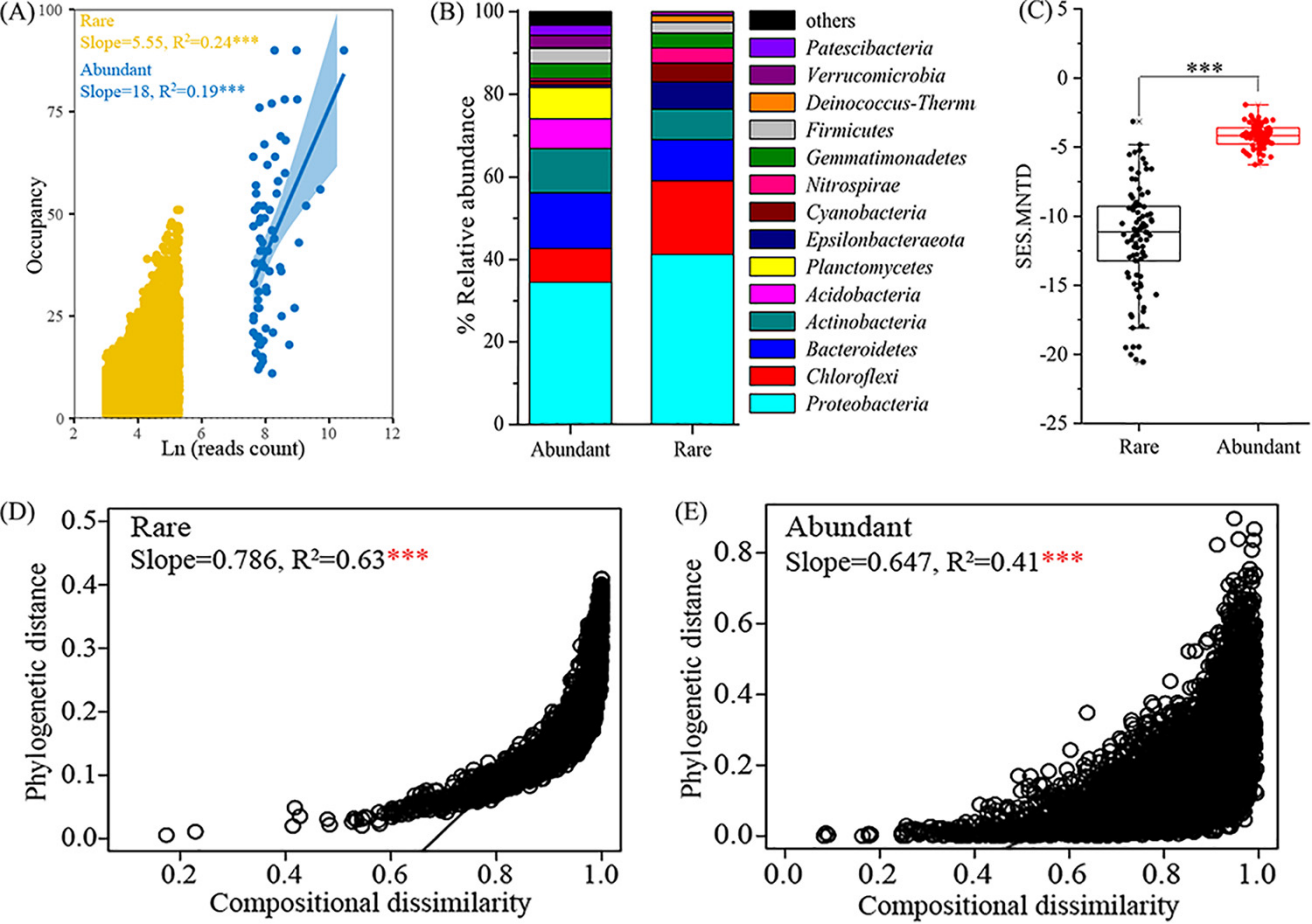
Taxonomic and phylogenetic diversities of rare and abundant bacteria in salinized agricultural soils. (A) Linear regressions showing abundance-occupancy relationships of rare and abundant bacterial OTUs. (B) Stacked column reflecting relative abundances of the top 14 phyla. (C) Box plot demonstrating difference in SES.MNTD values between rare and abundant bacteria. (D and E) Linear regressions exhibiting relationships between community compositional dissimilarity (Bray-Curtis dissimilarity) and phylogenetic distance (βMNTD) of rare (D) and abundant (E) bacteria. Asterisks represent significance (***, *P* < 0.001).

A total of 2,011,201 purified reads were classified into 13,449 OTUs. (OTUs with <20 reads were removed.) Rare bacteria comprised 84.8% of the total community richness (11,400 OTUs), and their total relative abundance accounted for 37.8% of the whole community. Abundant bacteria comprised 0.5% of the total community richness (70 OTUs), and their total relative abundance accounted for 14.5% of the entire community. Significantly positive correlations between occupancies and read numbers were found in both rare (*R*^2^ = 0.24, *P* < 0.001) and abundant (*R*^2^ = 0.19, *P* < 0.001) bacterial subcommunities (Fig. 1A). Abundant OTUs were more widely distributed than rare OTUs in salinized soils. Totals of 42.9% of abundant OTUs and 0.09% of rare OTUs were detected in more than 50% of all samples. Abundant OTUs were assigned to 11 phyla, while rare OTUs were assigned to 49 phyla. The abundant bacterial subcommunity was dominated by *Proteobacteria* (34.5%), *Bacteroidetes* (13.5%), *Actinobacteria* (10.7%), and *Chloroflexi* (2%), while the rare bacterial subcommunity was dominated by *Proteobacteria* (41.2%), *Chloroflexi* (17.8%), *Bacteroidetes* (10.0%), and *Actinobacteria* (7.38%) (Fig. 1B). These results indicated the distinct difference in distribution patterns between rare and abundant bacteria.

The taxonomic α-diversity represented by the Shannon-Wiener index was significantly higher in the rare bacterial subcommunity (5.22 to 7.51) than in the abundant bacterial subcommunity (0.86 to 3.29) (Wilcoxon rank sum test, *P* < 0.001). The taxonomic α-diversity of both rare and abundant bacteria was more significantly correlated with EC than other environmental factors (Table 1). The phylogenetic α-diversity (standardized index of effect size measure of mean nearest-taxon distance [SES.MNTD]) was dramatically lower for the rare than for the abundant bacteria, the values for which were all less than zero, and all *P* values were <0.05 (Wilcoxon rank sum test, *P* < 0.001 [Fig. 1C]). The phylogenetic α-diversity of rare bacteria was more significantly correlated with EC, and the phylogenetic α-diversity of abundant bacteria was more significantly correlated with total carbon (TC) (Table 1). Additionally, significant correlations between compositional dissimilarity and phylogenetic distance (β mean nearest-taxon distance [βMNTD]) were observed in rare (*R*^2^ = 0.63, *P* < 0.001 [Fig. 1D]) and abundant (*R*^2^ = 0.41, *P* < 0.001 [Fig. 1E]) bacterial subcommunities. These results indicated the distinct differences in taxonomic and phylogenetic diversities between rare and abundant bacteria.

**TABLE 1:** Pearson correlations between environmental factors and taxonomic (Shannon-Wiener index) and phylogenetic (SES.MNTD) α-diversities, as well as Mantel tests of environmental factors against phylogenetic turnover (βNTI)^*a*^

Environmental factor	Taxonomic α-diversity		Phylogenetic α-diversity		Community assembly	
	Rare	Abundant	Rare	Abundant	Rare	Abundant
pH	−0.253*	−0.239*	−0.120	0.024	0.126***	−0.098**
EC	−0.617**	−0.347**	0.399**	−0.190	0.387***	0.219***
TC	0.139	−0.014	0.050	0.304**	0.045**	−0.007
TN	0.370**	0.209*	−0.039	0.291**	0.156***	0.001
IP	0.043	−0.020	0.179	0.033	−0.060**	−0.022
OP	0.216*	0.098	0.065	0.033	−0.003	−0.012
TP	0.222*	0.083	0.144	0.046	−0.029*	−0.041**
AP	−0.209*	−0.197	−0.164	−0.047	0.070**	0.001
TK	0.428**	0.312**	−0.284**	0.044	0.228***	0.119***
AK	−0.078	−0.144	0.188	0.036	0.109***	0.128***
NH_4_	0.083	0.024	0.145	0.087	0.054**	0.025*
NO_3_	0.216*	0.070	−0.065	0.066	0.108***	0.093**

a The abbreviations of the environmental factors listed are defined in Materials and Methods. Asterisks denote significance levels (*, *P* < 0.05; **, *P* < 0.01; ***, *P* < 0.001).

**FIG 2:**
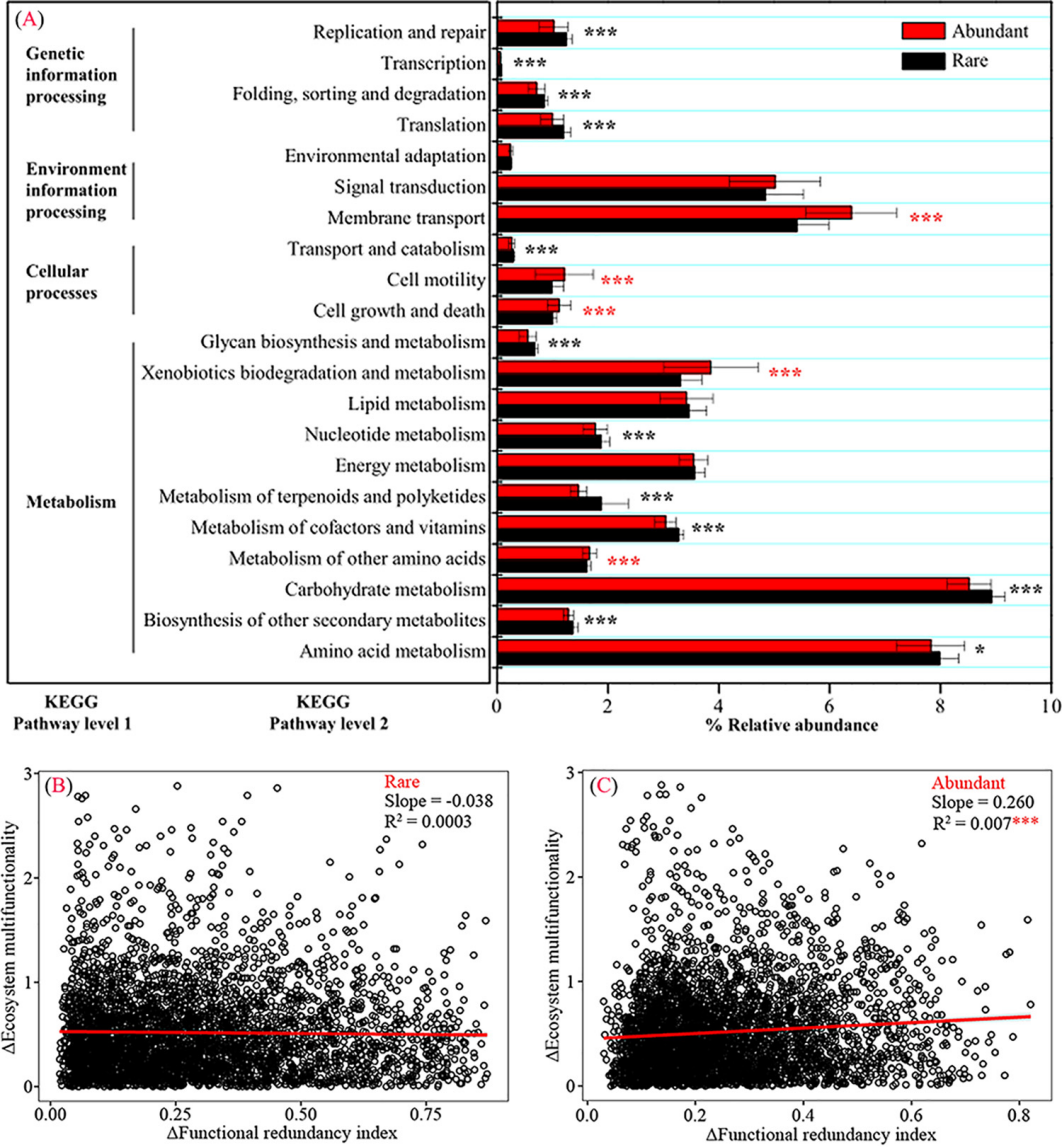
Functional differences in rare and abundant bacterial communities and contributions of bacterial functions to soil ecosystem multifunctionality. (A) Function profiling showing divergences in rare and abundant bacterial functions at KEGG pathway levels 1 and 2. (B and C) Linear regressions reflecting relationships between functional redundancy dissimilarity and ecosystem multifunctionality dissimilarity of rare (B) and abundant (C) bacteria. Asterisks represent significance (*, *P* < 0.05; ***, *P* < 0.001).

Based on functional profiling, 8,913 functions and 6,944 functions at KEGG pathway level 3 were found in rare and abundant bacterial subcommunities, respectively. Among these functions, 5,997 functions, including C-, N-, P-, and S-cycling-related enzymes or proteins exhibited higher functional redundancies in abundant bacterial subcommunity, whereas 2,916 functions showed higher functional redundancies in the rare bacterial subcommunity (see Fig. S2 in the supplemental material). Besides, 1,970 functions (e.g., methanol dehydrogenase [EC 1.1.1.244] and galactose oxidase [EC 1.1.3.9]) were exclusively in the rare bacterial subcommunity, and no function was unique in the abundant bacterial subcommunity. Salinity exhibited a significantly higher effect on bacterial functions compared to other environmental variables based on permutational multivariate analysis of variance (PERMANOVA) results (see Fig. S3 in the supplemental material). At KEGG pathway level 2, some functions (e.g., amino acid metabolism, nucleotide metabolism, translation, transcription, and replication and repair) were significantly higher in the rare than abundant bacterial subcommunity (Fig. 2A). Additionally, a significantly higher correlation between functional redundancy index (FRI) dissimilarity and EMF dissimilarity was found in the abundant bacterial subcommunity (*R*^2^ = 0.007, *P* < 0.001 [Fig. 2C]) than in rare bacterial subcommunity (*R*^2^ = 0.0003, *P* > 0.05 [Fig. 2B]). These results demonstrated that abundant bacteria had higher functional redundancies than rare ones, and abundant bacterial functions exhibited a larger contribution to soil EMF than rare ones.

10.1128/mSystems.01221-20.2 FIG S2 Differences in community function of rare and abundant bacteria at KEGG pathway level 3. A log ratio greater than 0 denotes that a function is more redundant in the red dot. Download FIG S2, DOCX file, 0.3 MB. Copyright © 2021 Wan et al. 2021 Wan et al. https://creativecommons.org/licenses/by/4.0/ This content is distributed under the terms of the Creative Commons Attribution 4.0 International license.

10.1128/mSystems.01221-20.3 FIG S3 Effects of environmental factors on community functions of rare and abundant bacteria based on PERMANOVA. Download FIG S3, DOCX file, 0.09 MB. Copyright © 2021 Wan et al. 2021 Wan et al. https://creativecommons.org/licenses/by/4.0/ This content is distributed under the terms of the Creative Commons Attribution 4.0 International license.

**FIG 3:**
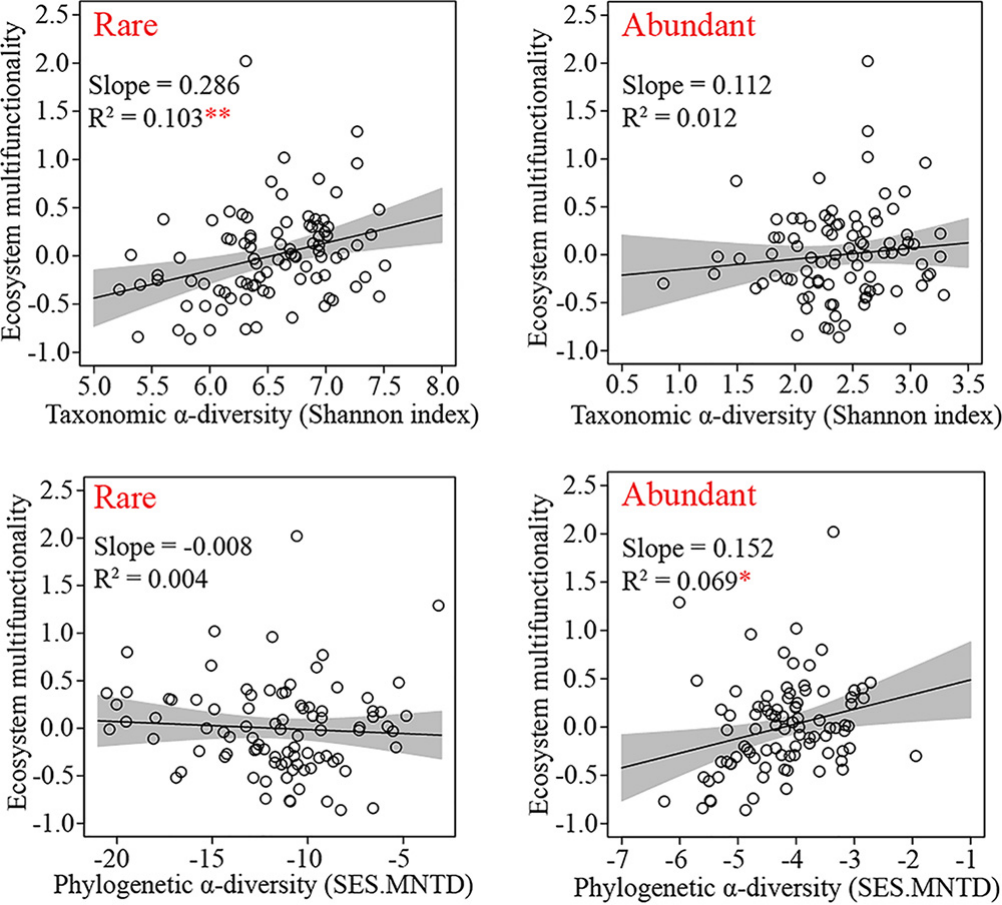
Linear regressions between soil ecosystem multifunctionality and taxonomic and phylogenetic α-diversities of rare and abundant bacteria. Asterisks denote significance (*, *P* < 0.05; **, *P* < 0.01).

The rare bacterial taxonomic α-diversity was significantly positively correlated with EMF (*R*^2^ = 0.103, *P* < 0.01), while abundant bacterial taxonomic α-diversity did not (*R*^2^ = 0.012, *P* > 0.05) (Fig. 3). In contrast, the abundant phylogenetic α-diversity was dramatically positively correlated with EMF (*R*^2^ = 0.069, *P* < 0.05), whereas rare phylogenetic α-diversity was slightly negatively correlated with EMF (*R*^2^ = 0.004, *P* > 0.05) (Fig. 3). These results revealed that taxonomic and phylogenetic α-diversities of rare and abundant bacteria contributed differently to EMF.

### Environmental responses of rare and abundant bacteria.

**FIG 4:**
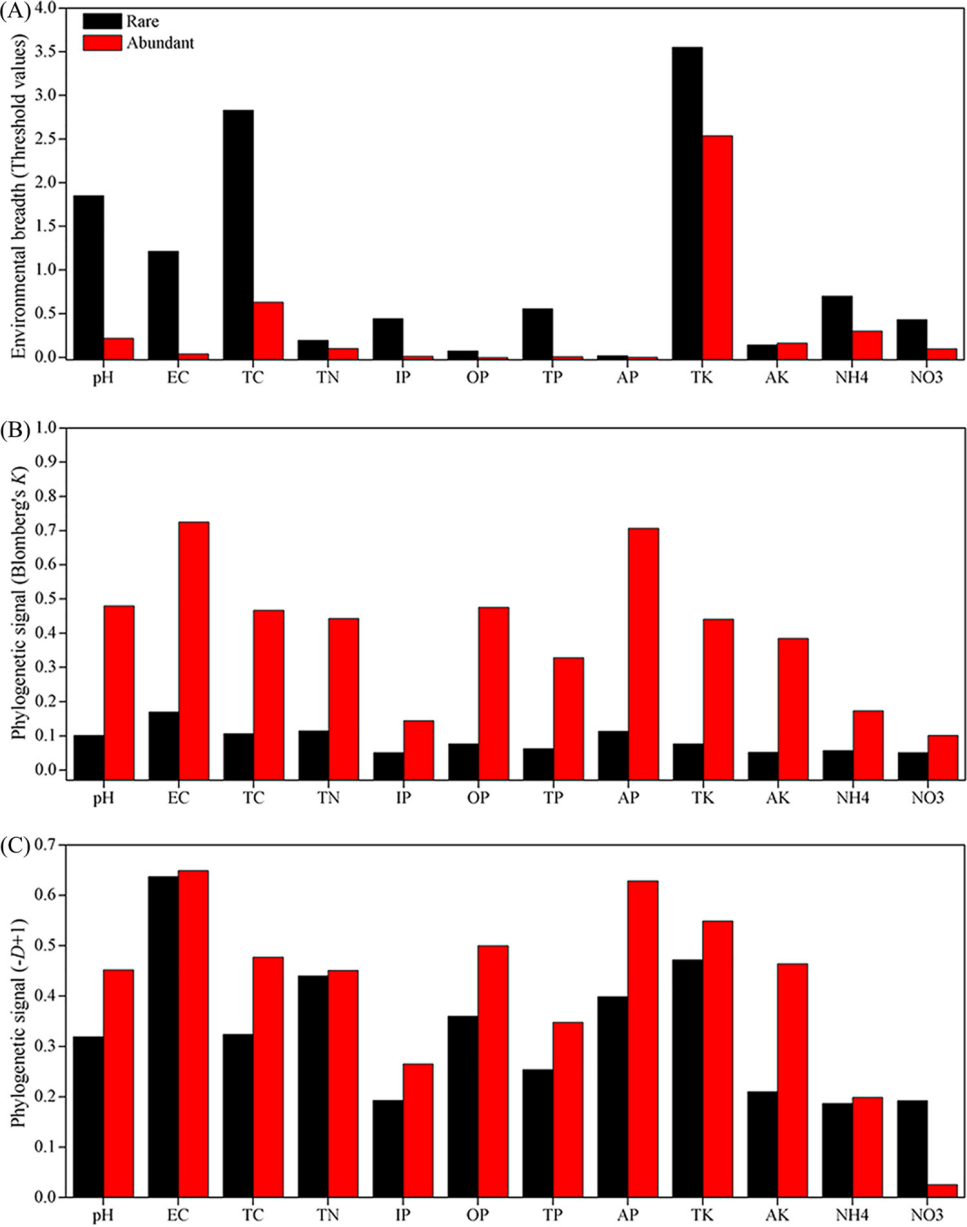
Environmental adaptation of rare and abundant bacteria in salinized agricultural soils. (A) Environmental breadth evaluated by the threshold values of rare and abundant taxa in response to environmental factors determined applying threshold indicator taxon analyses. (B and C) Phylogenetic signal reflecting the trait conservatism for environmental preferences of the rare and abundant bacteria employing Blomberg’s *K* statistic (B) and the Fritz-Purvis *D* test (C). The abbreviations of environmental factors are defined in Materials and Methods.

Environmental threshold analysis was applied to explore the responses of rare versus abundant bacteria to each of the tested environmental variables based on calculations of z+ (increasing taxa with increasing environmental gradient) and z− (decreasing taxa with increasing environmental gradient) (see Fig. S4 to S6 in the supplemental material). The rare bacteria showed a broader range of environmental thresholds compared to the abundant one for almost all tested variables (Fig. 4A). Abundant bacteria exhibited stronger phylogenetic signals for almost all tested environmental factors than the rare ones based on Blomberg’s *K* statistic (Fig. 4B) and Fritz-Purvis *D* test (Fig. 4C). The *Proteobacteria* validated such observations (see Fig. S7 in the supplemental material). For example, abundant *Proteobacteria* showed stronger phylogenetic signals for all tested environmental variables based on Blomberg’s *K* statistic and for 83.3% of the 12 environmental factors based on the Fritz-Purvis *D* test.

10.1128/mSystems.01221-20.4 FIG S4 Occurrence thresholds of rare and abundant bacteria with respect to environmental variables with significant effects on community composition: pH, EC (dS/m), TC (%), and TN (%). The Z-scores of all community members are shown. Yellow symbols show taxa decreasing with increasing environmental gradient (z−), while green symbols describe increasing taxa (z+). Download FIG S4, DOCX file, 0.5 MB. Copyright © 2021 Wan et al. 2021 Wan et al. https://creativecommons.org/licenses/by/4.0/ This content is distributed under the terms of the Creative Commons Attribution 4.0 International license.

10.1128/mSystems.01221-20.5 FIG S5 Occurrence thresholds of rare and abundant bacteria with respect to environmental variables with significant effects on community composition: IP (mg/g), OP (mg/g), TP (mg/g), and AP (mg/g). The Z-scores of all community members are shown. Yellow symbols show taxa decreasing with increasing environmental gradient (z−), while green symbols describe increasing taxa (z+). Download FIG S5, DOCX file, 0.5 MB. Copyright © 2021 Wan et al. 2021 Wan et al. https://creativecommons.org/licenses/by/4.0/ This content is distributed under the terms of the Creative Commons Attribution 4.0 International license.

10.1128/mSystems.01221-20.6 FIG S6 Occurrence thresholds of rare and abundant bacteria with respect to environmental variables with significant effects on community composition: TK (mg/g), AK (mg/g), NH_4_ (μg/g), and NO_3_ (μg/g). The Z-scores of all community members are shown. Yellow symbols show taxa decreasing with increasing environmental gradient (z−), while green symbols describe increasing taxa (z+). Download FIG S6, DOCX file, 0.5 MB. Copyright © 2021 Wan et al. 2021 Wan et al. https://creativecommons.org/licenses/by/4.0/ This content is distributed under the terms of the Creative Commons Attribution 4.0 International license.

10.1128/mSystems.01221-20.7 FIG S7 Phylogenetic signals of rare and abundant *Proteobacteria* for environmental preferences based on Blomberg’s *K* statistic and the Fritz-Purvis *D* test. Download FIG S7, DOCX file, 0.2 MB. Copyright © 2021 Wan et al. 2021 Wan et al. https://creativecommons.org/licenses/by/4.0/ This content is distributed under the terms of the Creative Commons Attribution 4.0 International license.

### Assembly processes in rare and abundant bacterial subcommunities.

**FIG 5:**
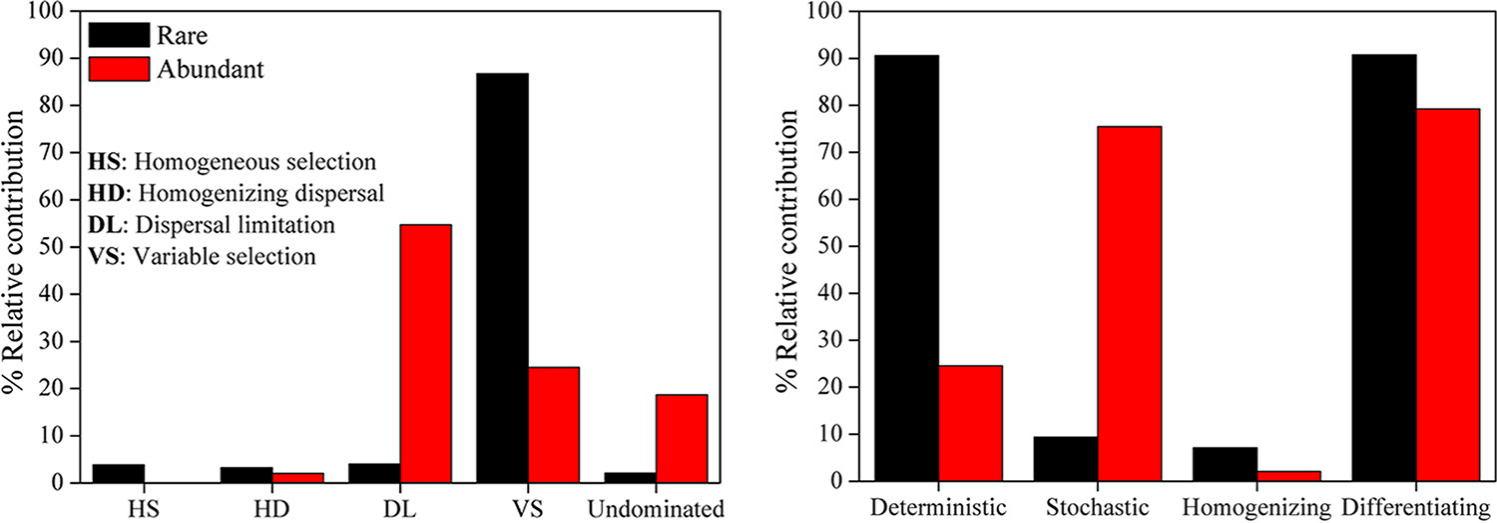
The relative contributions of ecological processes to the assemblies of rare and abundant bacterial subcommunities. Stochastic = Dispersal limitation + Homogenizing dispersal + Undominated processes; Deterministic = Variable selection + Homogeneous selection; Homogenizing = Homogeneous selection + Homogenizing dispersal; Differentiating = Variable selection + Dispersal limitation.

The relative contributions of assembly processes differed between rare and abundant bacterial subcommunities based on null model analysis (Fig. 5). Variable selection (86.7%) contributed most to rare bacterial community assembly, while dispersal limitation (54.7%) and variable selection (25.5%) contributed largely to abundant bacterial community assembly. Homogeneous selection, homogenizing dispersal, and “undominated” processes had limited effects on the assemblies of both rare and abundant bacterial subcommunities. Consequently, deterministic (90.6%) and differentiating (90.7%) processes dominated rare bacterial community assembly, whereas both stochastic (75.6%) and differentiating (79.2%) processes determined abundant bacterial community assembly.

Phylogenetic turnover represented by the β nearest-taxon index (βNTI) presented different correlations with changes in environmental variables based on results of Mantel tests (Table 1). More significant correlations were found between change in salinity and βNTI of both rare (*r* = 0.387, *P* < 0.001) and abundant (*r* = 0.219, *P* < 0.001) bacteria compared to other environmental factors. Additionally, pairwise comparisons of βNTI values for both rare and abundant bacteria were noticeably positively correlated with changes in salinity (see Fig. S8 in the supplemental material). This indicated that an increasing divergence in salinity resulted in a decrease in stochasticity in the assembly of the rare bacterial subcommunity, whereas an increasing difference in salinity led to an increase in the assembly of the abundant bacterial subcommunity. To further explore the links between salinity and βNTI, soil samples were rearranged into subgroups based on salinity. With increasing salinity, the relative contributions of stochasticity first decreased and then increased in the rare bacterial subcommunity and first increased and then decreased in the abundant bacterial subcommunity (see Fig. S9 in the supplemental material). These results suggested that salinity had significant effects on community assemblies of both rare and abundant bacteria.

10.1128/mSystems.01221-20.8 FIG S8 Linear regressions between βNTI of rare and abundant bacterial subcommunities and changes in soil salinity. Asterisks close to numbers denote significance level (***, *P* < 0.001). Download FIG S8, DOCX file, 0.4 MB. Copyright © 2021 Wan et al. 2021 Wan et al. https://creativecommons.org/licenses/by/4.0/ This content is distributed under the terms of the Creative Commons Attribution 4.0 International license.

10.1128/mSystems.01221-20.9 FIG S9 Patterns of βNTI across different categories in soil salinity for the rare and abundant bacteria subcommunities. Different letters above the column represent significance level (*P* < 0.05). Download FIG S9, DOCX file, 0.2 MB. Copyright © 2021 Wan et al. 2021 Wan et al. https://creativecommons.org/licenses/by/4.0/ This content is distributed under the terms of the Creative Commons Attribution 4.0 International license.

## DISCUSSION

Globally, about 1/10th of the total dry land surface on the earth to some degree suffers from salinization (40). Human activity and climate change accelerate soil salinization, which in turn threatens crop production and even forces human migration (12, 41). Soil salinization in China is severe, and approximately 3.67 × 10^7^ ha is saline-sodic soils (42). Restoration of salinized soils is challenging due to the complex biotic and abiotic characteristics of soils (43). In our viewpoint, evaluating soil EMF is important before performing restoration measures. Soil salinity as a crucial determinant affects microbial diversity and community composition (18, 44). In this work, we investigated the responses of rare and abundant bacteria to environmental factors and revealed relationships between EMF and rare and abundant bacterial diversities. Unexpectedly, we found differences in taxonomic and phylogenetic diversities, environmental breadth, phylogenetic signal, and community assembly processes between rare and abundant bacteria.

### Rare and abundant bacteria contributing differently to soil EMF.

Rare and abundant microorganisms show different effects on both nitrogen and phosphorus cycling (21, 45), which might contribute differently to soil EMF. However, only limited study has reported the interconnection between soil EMF and rare and abundant microbial diversities. Prior studies have reported that significant correlations are found between EMF and phylogenetic α-diversity of both bacteria and fungi in long-term fertilized soils (6) and between EMF and taxonomic α-diversity of microorganisms (i.e., archaea, bacteria, fauna, and fungi) in the northeastern and central Tibetan Plateau in China (4). In this study, we found that taxonomic α-diversity of rare bacteria and phylogenetic α-diversity of abundant bacteria showed significant effects on EMF in salinized agricultural soils. Meanwhile, taxonomic α-diversity of abundant bacteria and phylogenetic α-diversity of rare bacteria had little influence on soil EMF. This phenomenon might be due to difference in diversity maintenance between rare and abundant bacteria in salinized soils.

Previous literature has reported that salinity showed distinct effects on bacterial functions based on functional profiling (17). In our study, we found that the number of unique functions of rare bacteria was higher than that of abundant bacteria, while the number of abundant bacterial functional redundancies was comparably higher than that of rare bacteria. Consequently, abundant bacterial functions contributed significantly to soil EMF, while rare bacteria did not. These findings are reasonable because abundant bacteria present higher competition potential and growth rate than rare taxa (38, 39).

### Distinct differences in environmental adaptation at the taxonomic and phylogenetic levels.

Many studies have investigated distribution patterns and effects of environmental factors on community composition of rare and abundant bacteria (24, 36), despite their adaptations to the environment. We have attempted to provide insights into the responses of rare and abundant bacteria to ongoing environmental change in salinized agricultural soils. Here, environmental adaptations of rare and abundant bacteria were evaluated using two different measures: environmental breadth at taxonomic level based on TITAN (threshold indicator taxon) analysis (25, 46) and phylogenetic signal at phylogenetic level based on Blomberg’s *K* statistic and the Fritz-Purvis *D* test (47).

First, we found that rare bacteria presented broader response thresholds to almost all environmental variables compared to abundant bacteria. Prior study has reported that abundant fungi showed broader environmental breadths than rare fungi in Chinese agricultural soils (48). This is opposite to our hypothesis, and this divergence might be greatly attributed to the higher richness of rare contrary to abundant taxa. Besides, abundant bacteria were omnipresent rather than the rare ones in salinized agricultural soils, which is in line with several prior findings (20, 24, 49). In contrast, rare bacterial taxa were not distributed evenly, and most occurred only in a few soil samples, which agrees with previous reports (20, 24, 49). This phenomenon might be driven by rapid environmental changes (e.g., precipitation). A prior study has reported that soil salinity is coupled with water availability (18), and the precipitation could change soil salinity by washing away ions. This may facilitate the rapid adaptation of the rare bacteria, which shows some unique functions and relatively close phylogenetic clustering. Microbes adapting to environment changes have been clarified at both physicochemical and genetic levels (50). Prior research has indicated that a bacterial consortium containing *Corynebacterium* and *Petrimonas* exhibited good performance for degrading phenol under the condition of 30 mg/liter NaCl (51). In addition, Bacillus subtilis adapts to high-salinity shock by adjusting the DegS/DegU and SigW transcription level (52). Environmental threshold analysis applying TITAN has been reported in some terrestrial microbial diversity-related studies (25, 32, 53). For instance, arbuscular mycorrhizal fungi exhibited broad environmental thresholds to nitrogen and phosphorus deposition (53). The findings of environmental breadths of microbes employing TITAN analysis are attractive, but are also controversially discussed considering implications to the real field situation. Therefore, confirmatory experiments are required to test such statistical results before performing environmental policy. Nevertheless, our results offer a solid statistical hint for the potentially broader environmental breadths of rare bacteria than abundant bacteria in salinized agricultural soils. This might explain why rare rather than abundant bacterial taxonomic α-diversity presented a significant effect on soil EMF. These findings are, to our knowledge, novel and have not been reported before.

Second, we found that abundant bacteria showed stronger phylogenetic signals for environmental preferences than rare bacteria based on both Blomberg’s *K* statistic and the Fritz-Purvis *D* test. The results are in line with prior studies describing that abundant taxa showing stronger phylogenetic signal than rare taxa (20), which might signify that closely correlated microbial taxa present more similar ecological preferences across environmental gradients within the abundant taxa. It has been reported that microbial functional traits based on the ecological preferences mainly rely on microbial evolutionary history (54, 55). For example, evolutionary history has stronger effects on bacterial functional traits than environmental heterogeneity (e.g., precipitation and temperature) (56). In addition, the microbial response trait of salinity preference is reported to be deeply phylogenetically conserved (16, 26), which is related to the critical role of salinity in determining microbial community structure (11, 18). In the present study, abundant bacteria showing stronger phylogenetic signals for ecological preferences might reflect that abundant bacteria had more phylogenetic niche conservatism with respect to the evolutionary history of environmental adaptation (57). Unexpectedly, tighter connections (larger *R*^2^ values in Fig. 1D and E) between phylogenetic distance and community compositional dissimilarity were found in rare than abundant bacteria. This might be mainly due to the phylogenies of abundant bacteria being less sensitive to ongoing environmental changes (24). The potential to preserve a community’s phylogeny could reflect the ability of the community to maintain the ecological niche (58). Thereby, the decoupling between bacterial community composition and phylogenetic distance reveals that abundant bacteria are relatively better at preserving ecological niches than rare bacteria, which agrees with the finding of a stronger phylogenetic signal of abundant bacteria than rare bacteria. These findings mentioned above might explain why abundant rather than rare bacterial phylogenetic α-diversity exhibited a noticeable effect on soil EMF.

### Different assembly processes of rare and abundant bacterial subcommunities.

Disentangling ecological processes underlying community assembly is a key issue in microbial ecology (18, 31, 33, 37). In the present study, deterministic processes (i.e., variable selection) dominated rare bacterial community assembly, which is not in accordance with a prior report describing that homogeneous selection dominated rare bacterial community assembly in agricultural soils (20). Abundant bacterial community assembly was mainly determined by stochasticity (mainly dispersal limitation), which is not in line with a previous report showing that homogeneous selection presents a crucial effect on abundant bacterial community assembly (36). Besides, abundant bacterial community assembly was more affected by stochasticity than rare bacterial community assembly, which disagrees with findings reporting that the rare bacterial community is more strongly influenced by stochasticity than the abundant one in a salt marsh terrestrial ecosystem (35), in Tibetan Plateau grassland soils (24), and in rice paddy soils (36). These divergences might be attributed to differences in environmental heterogeneity and the ability of individual species to deal with environmental changes (20). Compared to soils collected from natural wetlands and paddy fields (24, 35, 36), our soils were collected from salinized abandoned farmlands. Stochastic processes increase under relatively-high-nutrient conditions, while deterministic processes seem to be more related to low-nutrient conditions (31). Besides, rare taxa are more specialists, and abundant taxa are more generalists (59). The high richness of rare taxa shows a high spectrum of specialist species that are handling quite well conditions in different habitats—hence, their high ecological breadth (22).

Based on pairwise community comparison applying null model analysis, we found the βNTI of both rare and abundant bacterial subcommunities to be more closely correlated with soil salinity than other environmental variables. This might imply that soil salinity is the crucial factor adjusting the balance between deterministic and stochastic processes for assemblies of both rare and abundant bacterial subcommunities in salinized agricultural soils. The critical role of soil salinity in determining both rare and abundant bacterial community assemblies might be partially attributed to the toxicity of salinity to the microbial cell. High salinity leads to high osmotic pressure for cells, which in turn decreases cell metabolic activity (11, 13, 14, 44, 60). Considering the contributions of ions (e.g., sulfate and chloride) to salinity, other environmental variables might also partially adjust the balance between deterministic and stochastic processes in community assemblies of both rare and abundant bacteria. Future work will explore more environmental variables affecting community assembly of rare and abundant bacteria in more different terrestrial ecosystems with a broader salinity range.

**FIG 6:**
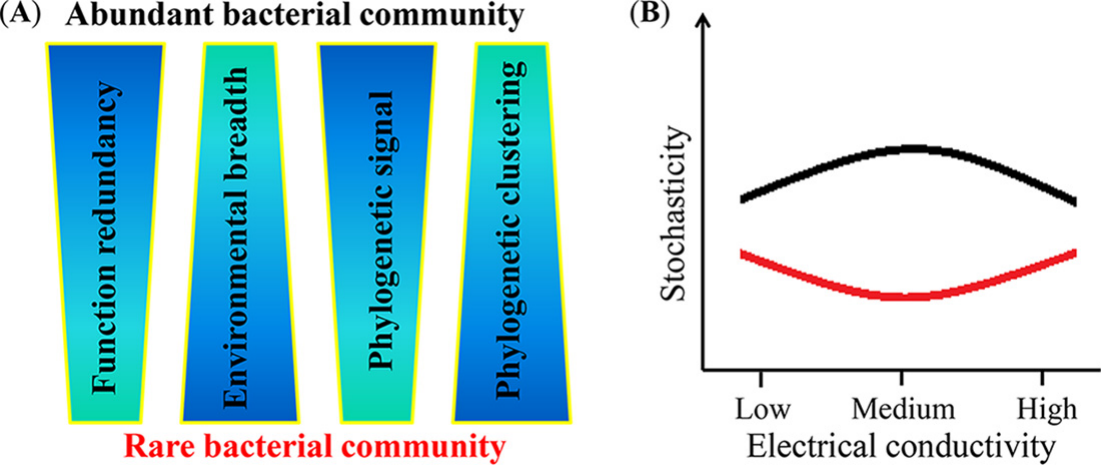
Conceptual models revealing (A) environmental response and (B) stochasticity in the assemblies of rare and abundant bacterial subcommunities under the influence of soil salinity.

Ultimately, a conceptual paradigm was constructed to reveal differences in rare and abundant bacteria in salinized agricultural soils (Fig. 6). Abundant bacteria possessed higher functional redundancies and stronger phylogenetic signals of ecological preferences than rare ones, while rare bacteria exhibited closer phylogenetic clustering and broader environmental breadths than abundant ones. Soil salinity was the crucial factor in determining community assembly of rare and abundant bacteria, showing distinct changes in stochasticity with higher salinity.

In conclusion, we characterized taxonomic and phylogenetic α-diversities, environmental breadths, phylogenetical signals, and community assembly processes of rare and abundant bacteria along environmental gradients in salinized agricultural soils. To our knowledge, our DNA-based data sets and statistical analysis are the first to reveal that taxonomic and phylogenetic α-diversities of rare and abundant bacteria contribute differently to soil EMF, and functional redundancies of abundant bacteria rather than rare bacteria contribute significantly to EMF. Rare bacterial community assembly was dominated by deterministic processes, and abundant bacterial community assembly was determined by stochastic processes. Soil salinity exhibits a critical role in mediating the balance between deterministic and stochastic processes in community assemblies of both rare and abundant bacteria. Our findings are of importance for understanding the maintenance of bacterial diversity and bacterial contribution to soil EMF and offer a way to predict the responses of bacteria to environmental changes in salinized soils.

## MATERIALS AND METHODS

### Site description and soil collection.

Yingcheng City, located in Hubei Province, in the middle of China (113°21′E, 30°55′N), is famous for its huge salt resources (mainly NaCl and Na_2_SO_4_). Many salt mines have been exploited to provide chemical plants with brine for synthesis of chemical products (e.g., NH_4_Cl, Na_2_CO_3_, and urea). Farmlands near factories presented different degrees of salinization due to unreasonable mining (near farmland), careless maintenance of iron pipe (where leakage always happens), irrational discharge of untreated wastewater, and irrigation with saline groundwater.

Soils were collected from farmlands in 1 to 4 May 2019; the sampling sites are shown in Fig. S1 in the supplemental material. Soil samples were collected at a depth of 0 to 15 cm by using a hand core probe, and 9 soil cores from each site were placed into one sterile plastic bag and regarded as one sample. The 90 soil samples were delivered to the laboratory within 24 h after sampling and stored at 4°C.

10.1128/mSystems.01221-20.1 FIG S1 Map of 90 soil sampling sites. Download FIG S1, DOCX file, 0.9 MB. Copyright © 2021 Wan et al. 2021 Wan et al. https://creativecommons.org/licenses/by/4.0/ This content is distributed under the terms of the Creative Commons Attribution 4.0 International license.

### Determination of soil physicochemical properties and enzyme activity.

The 90 soil samples were sieved through a 0.85-mm sieve and freeze-dried. We determined soil physicochemical properties, including electrical conductivity (EC), pH, total carbon (TC), total nitrogen (TN), NH_4_^+^-N, NO_3_^−^-N, total phosphorus (TP), available phosphorus (AP), inorganic phosphorus (IP), organic phosphorus (OP), total potassium (TK), and available potassium (AK). Detailed descriptions of determination approaches were reported in previous studies (37, 61).

Here, we selected multiple functional enzymes to reflect soil microbial activity (37), including the C-cycling-related enzymes β-1,4-glucosidase (βG [EC 3.2.1.21]), β-d-fructofuranoside hydrolase (βF [EC 3.2.1.26]), β-d-cellobiohydrolase (CBH [EC 3.2.1.91]), and phenol oxidase (PhOx [EC 1.10.3.2]), the N-cycling-related enzymes arylamidase (Ary [EC 3.4.11.2]), β-1,4-*N*-acetyl-glucosaminidase (NAG [EC 3.2.1.30]), and urease (EC 3.5.1.5), and the P-cycling-related enzymes alkaline phosphatase (Pho [EC 3.1.3.1]) and phytase (Phy [EC 3.1.3.8]). The appropriate substrates and agents employed for measurement of enzyme activities were summarized in a prior study (37).

We applied 17 ecosystem functions, including C-cycling-related functions (TC, βG, CBH, βF, and PhOx), N-cycling-related functions (TN, NH_4_^+^-N, NO_3_^−^-N, urease, Ary, and NAG), and P-cycling-related functions (TP, Olsen P, IP, OP, Pho, and Phy) to calculate the EMF index by employing Z-score transformation (5, 6).

### DNA extraction, Illumina sequencing, and sequence processing.

The total genomic DNA was extracted from 0.5 g of freeze-dried soil according to a standardized DNA extraction method (ISO-11063) (62). The extracted DNA was purified by removing potential humic substances and enzyme inhibitors and applying a DNA-EZ Reagents M Humic acid-Be-Gone B kit (Sangon Biotech, Shanghai, China). The DNA concentration was measured by employing a NanoDrop 2000 spectrophotometer (Thermo Fisher Scientific, Waltham, MA, USA). All extracted DNA samples were stored at −80°C.

The universal primers 338F (5′-ACT CCT ACG GGA GGC AGC A-3′) and 806R (5′-GGA CTA CHV GGG TWT CTA AT-3′) were used to amplify the 16S rRNA gene (63). PCR was conducted in triplicate, applying a thermal cycler (ABI 9700; Thermo, USA), and performed under the following conditions (20 μl): an initial denaturation at 95°C for 3 min, followed by 30 cycles of 95°C for 40 s, 58°C for 40 s, and 72°C for 50 s, and then a final extension at 72°C for 10 min. The triplicate PCR products were pooled and purified by gel electrophoresis and extracted by employing an AxyPrep DNA gel extraction kit (Axygen, Hangzhou, China). Sequencing was performed on an Illumina MiSeq platform at Personal Biotechnology Co., Ltd. (Shanghai, China).

The raw reads were purified following the pipeline of QIIME1.8.0 and with the help of FLASH (64). To minimize the influences of random sequencing errors, we removed (i) sequences that did not exactly match barcodes and primers, (ii) sequences with maximum homopolymers that were less than 10 bp, (iii) sequences with an average quality score of less than 20, and (iv) sequences that contained ambiguous base calls (11). The purified sequences were clustered into operational taxonomic units (OTUs) by using UCLUST at a 97% similarity level against the SILVA v132 reference. We used the FastTree tool to build a phylogenetic tree based on the 16S rRNA gene sequences of representative OTUs (65).

### Data analysis.

To avoid random effects on the identification of rare taxa, OTUs that contained less than 20 reads were removed (20). The rare and abundant OTUs were defined following recent reports (20, 38). Briefly, OTUs with relative abundances of <0.01% of the total sequences were regarded as “rare,” those with relative abundances of >0.1% were designated “abundant,” and the rest (0.01 to 0.1%) were defined as “intermediate.”

To perform functional profiling of rare and abundant bacterial subcommunities, the functional redundancy index (FRI) of each sample was calculated based on sequence similarity of the 16S rRNA gene by applying the ‘‘Tax4Fun2” package in R (66). The FRI is based on the proportion of species capable of performing a particular KEGG function and their phylogenetic relationship to each other (66). We compared differences in function between rare and abundant bacterial subcommunities at KEGG pathway levels 1, 2, and 3. Permutational multivariate analysis of variance (PERMAVONA) was applied to evaluate the influences of different environmental variables on functions of rare and abundant bacteria by employing the “adonis” function in the “vegan” package of R.

To assess the phylogenetic clustering of rare and abundant taxa, the standardized index of effect size measure of the mean nearest-taxon distance (SES.MNTD) was computed based on the null model, applying the “ses.mntd” function in the “picante” package of R (67). To estimate the pairwise phylogenetic distance between communities, the β mean nearest-taxon distance (βMNTD) was calculated using the “comdistnt” function in the “picante” package (67).

To compute the threshold values of rare and abundant taxa in response to each environmental factor described above, threshold indicator taxon analysis (TITAN) was performed applying the “TITAN2” package in R (46). The sums of taxon scores for OTUs were applied to determine upper and lower thresholds of difference in the rare and abundant subcommunities based on environmental variables. In addition, we determined potential trait information about rare and abundant bacteria. Briefly, the ecological preferences for each OTU were determined by calculating Spearman correlations between environmental variables and relative abundances of microbial taxa (68). The OTUs positively or negatively correlated with salinity were designated “high-salinity-preferred” or “low-salinity-preferred”; the OTUs positively or negatively correlated with pH were designated “alkaline-preferred” and “acid-preferred.” Subsequently, we employed Blomberg’s *K* statistic and the Fritz-Purvis *D* test to determine the phylogenetic signals for the environmental preferences of rare and abundant taxa (47). The Blomberg’s *K* statistical analysis describes a phylogenetic signal that compares the observed signal in a trait to the signal under a Brownian motion-based metric of trait evolution on a phylogeny (69). The *K* value was determined by employing the “multiPhylosignal” function in the “picante” package of R (67), where *K* values closer to 0 represent a convergent or random pattern of evolution, and *K* values higher than 1 reflect strong phylogenetic signals and conservatism of traits (47). We computed the phylogenetic signal of binary traits applying the “phylo.D” function in the “caper” package of R (70). The Fritz-Purvis phylogenetic dispersion (*D*) value compares the observed sister clade differences in the trait against those expected for a random phylogenetic pattern (47, 70). The *D* value was transformed into −*D *+ 1 to compare with Blomberg’s *K* statistic (47). The evolution of a study trait (i) does not denote a noticeable signal when −*D *+ 1 = 0 and (ii) is more conserved than expected by chance when −*D *+ 1 > 0.

Rare and abundant bacterial community assembly processes were evaluated by applying null model in the “picante” package in R (20, 31, 33). Briefly, the β nearest-taxon index (βNTI) and null-model-based Bray-Curtis-based Raup-Crick (RC_bray_) were employed to calculate the differences in phylogenetic and taxonomic diversities. If|βNTI| is >2, it represents the dominance of deterministic processes, with significantly more (i.e., variable selection; βNTI > 2) and less (i.e., homogeneous selection; βNTI < −2) phylogenetic turnover than expected. When |βNTI| is <2, RC_bray_ < −0.95 and RC_bray_ > 0.95 denote the relative contributions of homogenizing dispersal and dispersal limitation, respectively. The remaining category of|βNTI| < 2 and |RC_bray_| < 0.95 indicates the contribution of “undominated” assembly, which mostly contains ecological drift, diversification, weak selection, and/or weak dispersal (32, 33). Homogenizing processes were estimated as the sum of homogenizing dispersal and homogeneous selection, and the differentiating processes were evaluated as the sum of dispersal limitation and variable selection. The correlations between βNTI and physicochemical variables were determined using Mantel’s tests, where the dissimilarities in physicochemical variables applied Euclidean distance.

### Data availability.

All sequencing data associated with this study have been deposited in the NCBI (http://www.ncbi.nlm.nih.gov) Sequence Read Archive (SRA) database under accession no. PRJNA629647.
