# Supplementary figures and images for: Bridging Rare and Abundant Bacteria with Ecosystem Multifunctionality in Salinized Agricultural Soils: from Community Diversity to Environmental Adaptation
Source: mSystems. 2021 Mar 30;6(2):e01221-20. doi: 10.1128/mSystems.01221-20 (PMC8547000; doi:10.1128/mSystems.01221-20)

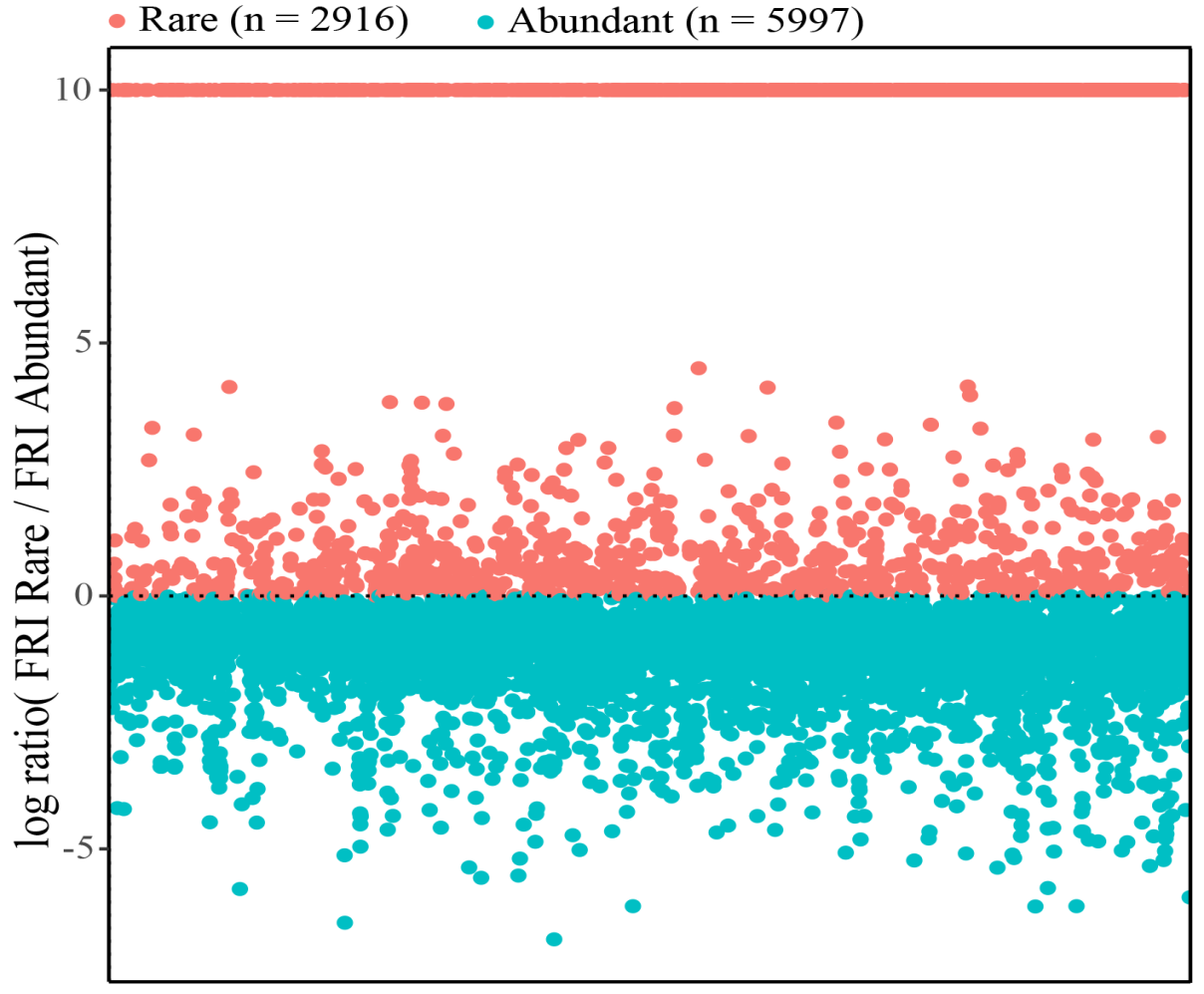

Supplement: FIG S2 [file msystems.01221-20-sf002.docx]

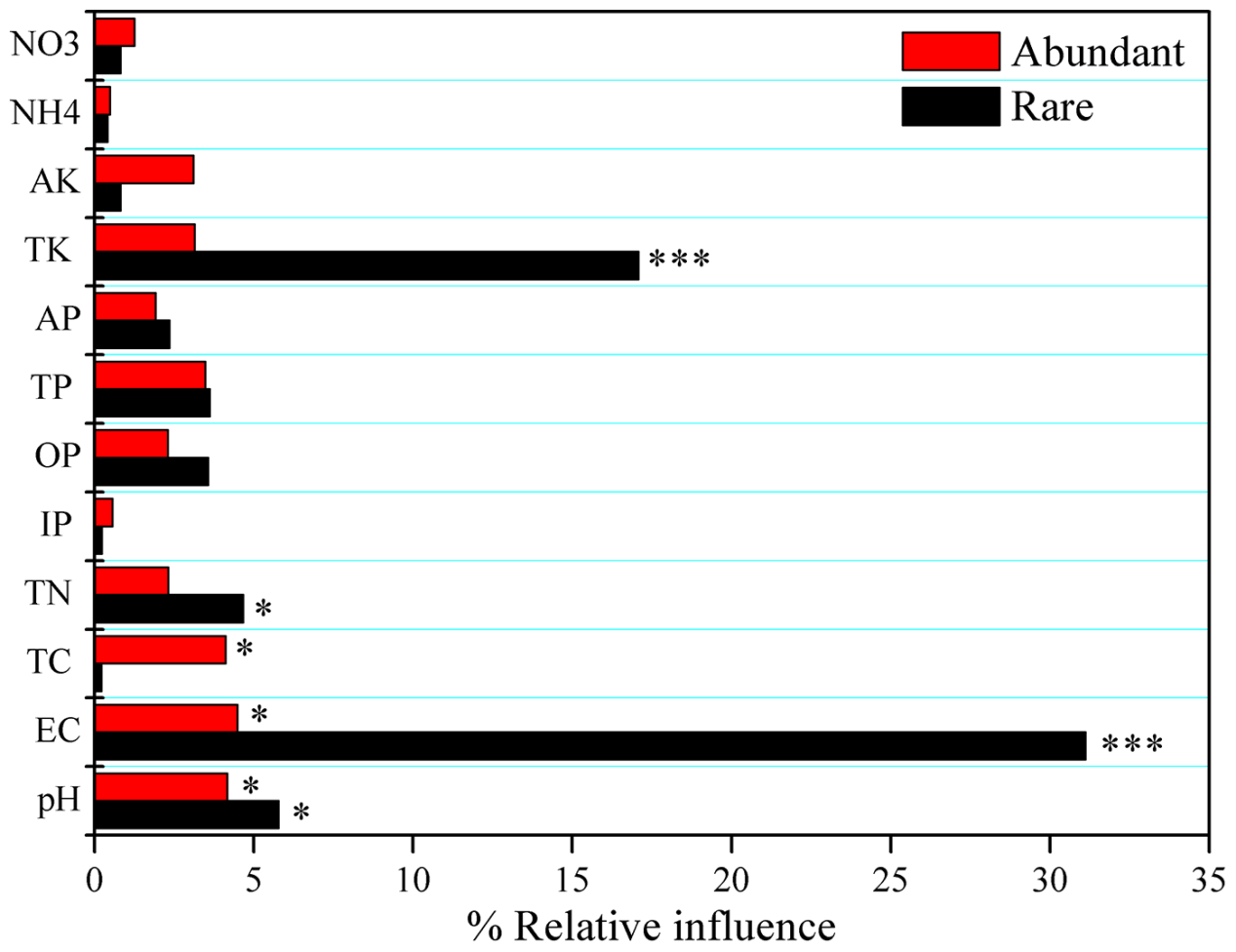

Supplement: FIG S3 [file msystems.01221-20-sf003.docx]

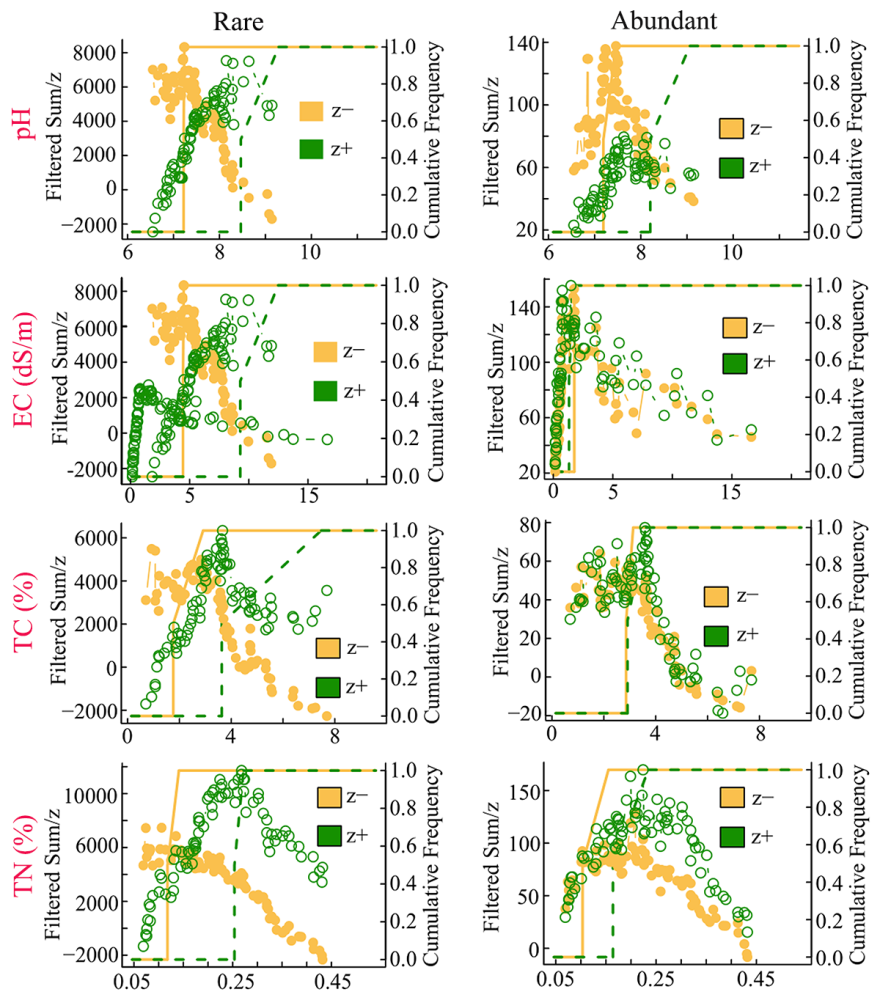

Supplement: FIG S4 [file msystems.01221-20-sf004.docx]

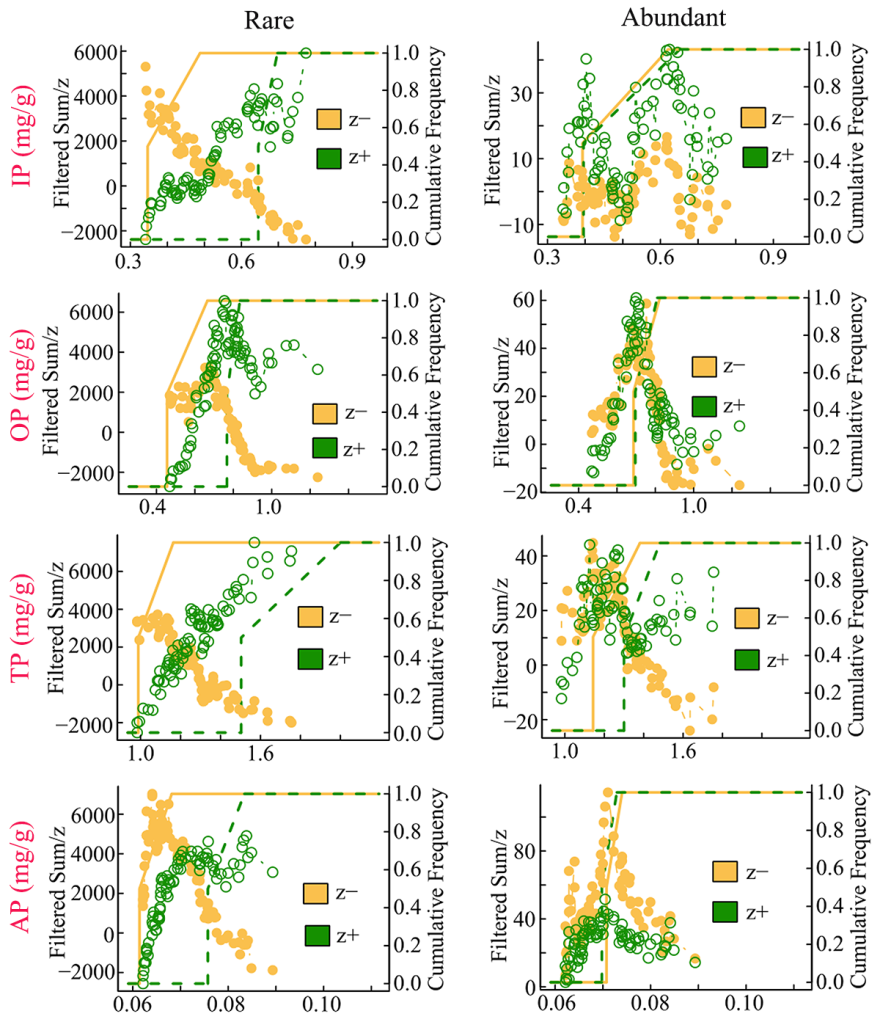

Supplement: FIG S5 [file msystems.01221-20-sf005.docx]

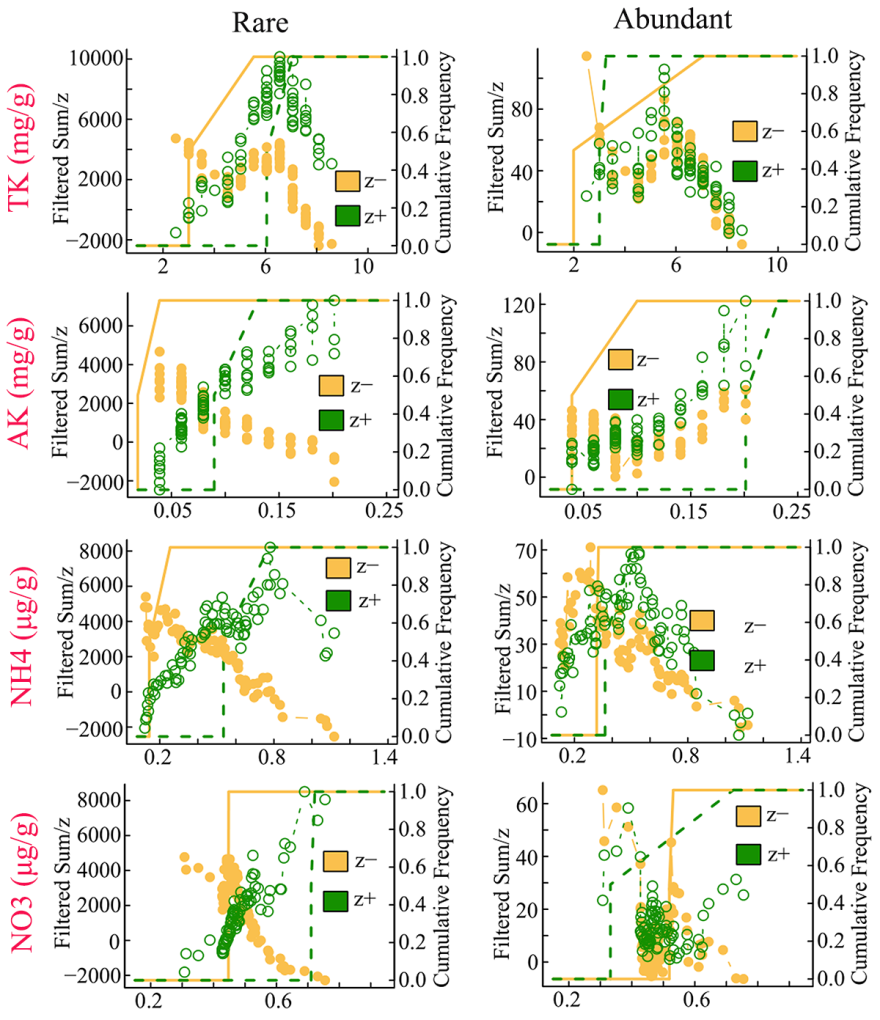

Supplement: FIG S6 [file msystems.01221-20-sf006.docx]

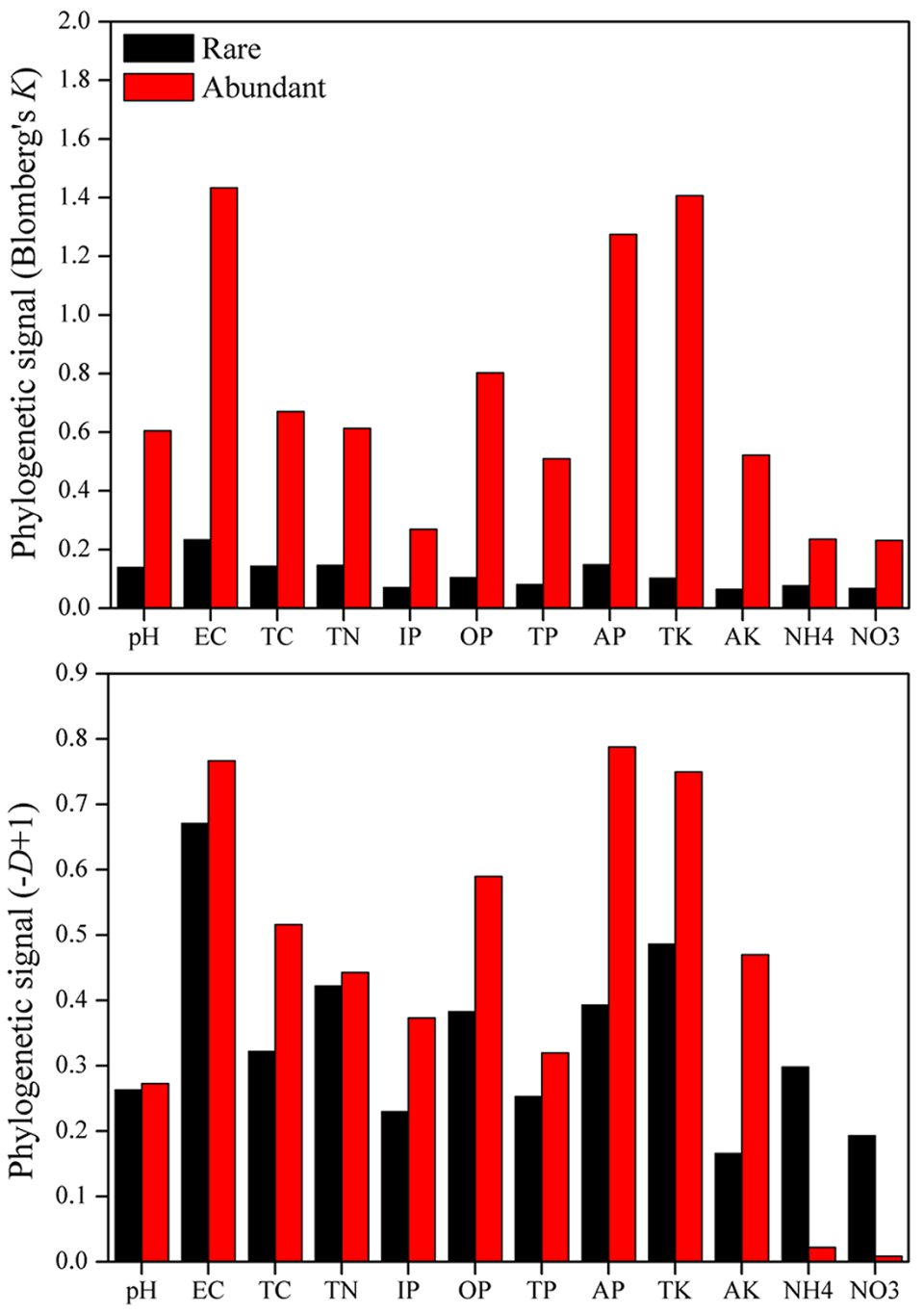

Supplement: FIG S7 [file msystems.01221-20-sf007.docx]

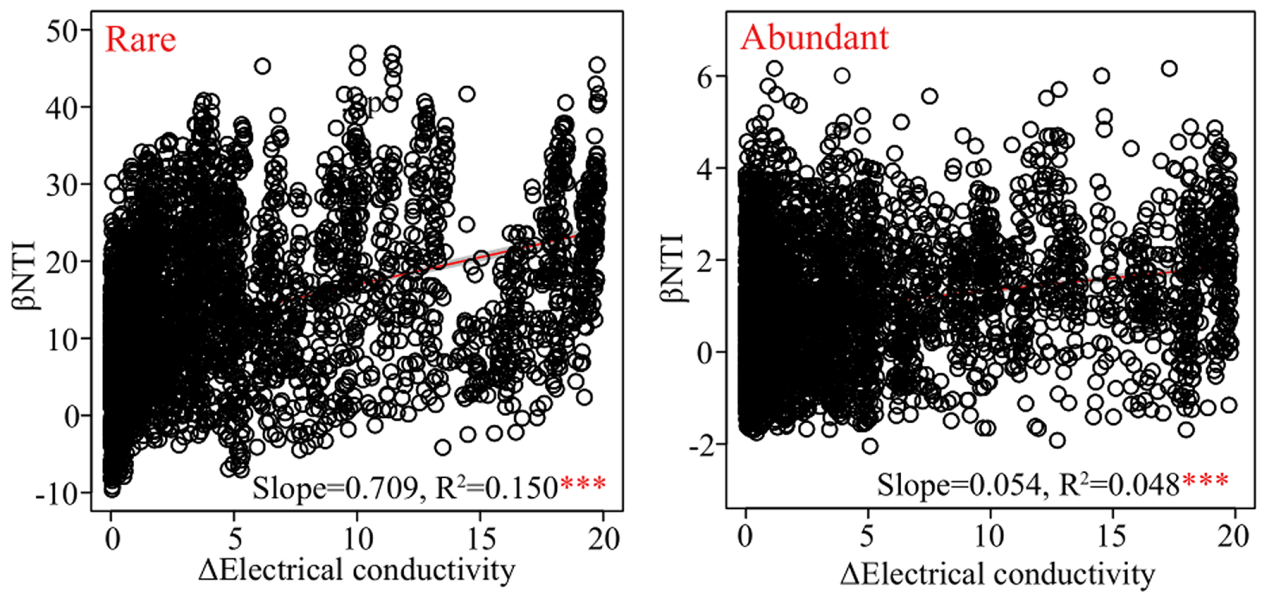

Supplement: FIG S8 [file msystems.01221-20-sf008.docx]

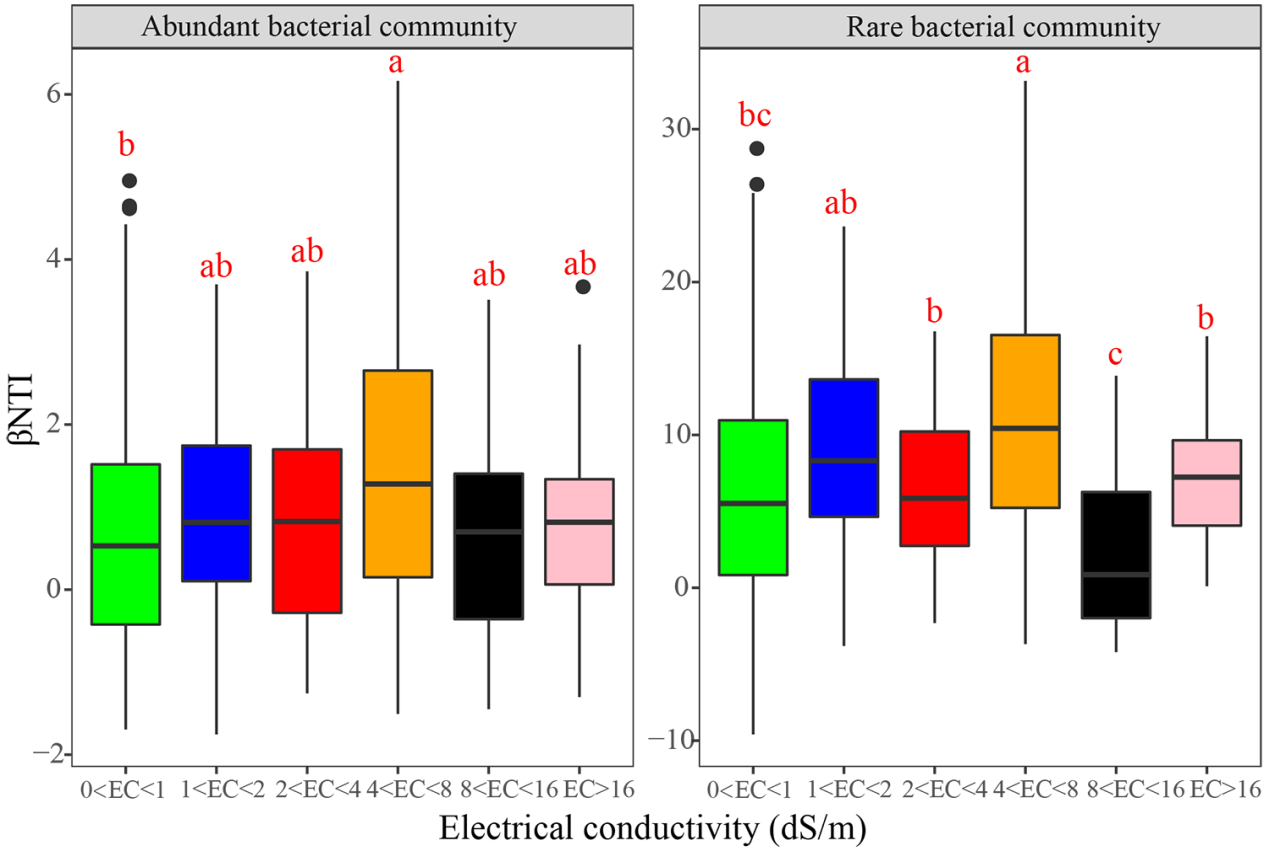

Supplement: FIG S9 [file msystems.01221-20-sf009.docx]

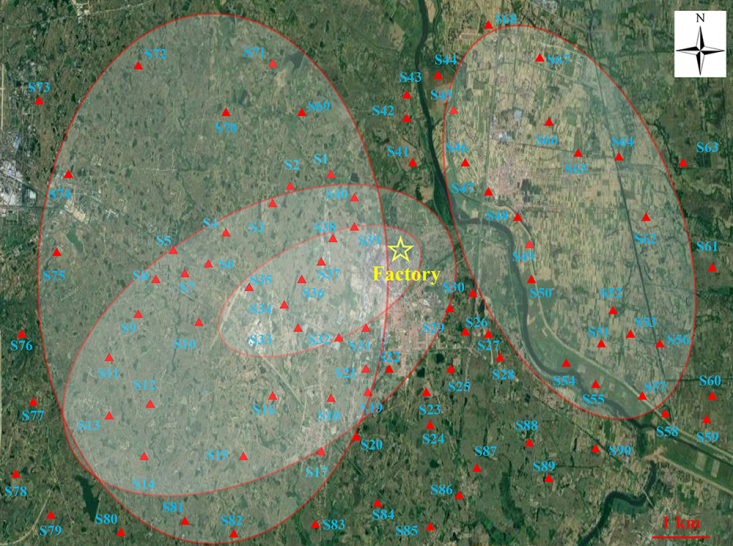

Supplement: FIG S1 [file msystems.01221-20-sf001.docx]
